# Hyponatremia and rhabdomyolysis from excessive water intake for ureteral stone expulsion in a patient with mental disorder: A case report

**DOI:** 10.1097/MD.0000000000044214

**Published:** 2025-09-05

**Authors:** Xiaowen Gao, Jian Ji, Yafei Wang, Tiancan Yang, Lingmin Lei, Lvyang Chen, Yuanquan Zhu

**Affiliations:** a Department of Urology, The Third People’s Hospital of Yunnan Province, The Second Affiliated Hospital of Dali University, Kunming, China.

**Keywords:** hypokalaemia, hyponatremia, mental disorder, rhabdomyolysis, ureteral stones, water intoxication

## Abstract

**Rationale:**

Primary polydipsia refers to excessive water intake due to psychogenic or non-psychogenic causes without being secondary to conditions such as hyperglycemia or renal dysfunction. Most cases of primary polydipsia are psychogenic in nature, with few cases of non-psychogenic primary polydipsia reported in the literature. In this case, the patient’s excessive water intake appeared to be influenced by both psychogenic and non-psychogenic factors.

**Patient concerns:**

A 43-year-old male patient with a history of psychiatric illness was diagnosed with ureteral stones and was engaged in excessive water intake in an attempt to facilitate stone passage. Subsequently, he developed facial and bilateral lower extremity edema. On physical examination, the patient appeared anxious, exhibited a depressed mood, and demonstrated poor speech, but remained fully conscious. Physical examination revealed facial and bilateral lower extremity edema with tenderness and percussion pain in the left renal region. Neurological examination and other systemic evaluation revealed no significant abnormalities. Laboratory tests revealed hyponatremia, hypokalemia, and markedly elevated creatine kinase levels.

**Diagnoses:**

The diagnosis of hypervolemic hypotonic hyponatremia complicated by rhabdomyolysis was established based on the patient’s clinical manifestations and laboratory findings.

**Interventions:**

Furosemide was initiated on day 2 for diuresis and transitioned to continuous micropump infusion for precise dosing. Persistent edema prompted albumin administration (day 4) to address hypoalbuminemia and low-dose dopamine for administration renal perfusion. The psychiatric symptoms were managed with quetiapine and sertraline. Urine culture on day 6 confirmed urinary tract infection, prompting cefoperazone therapy. By day 10, the edema had resolved, and ureteroscopic lithotripsy was performed.

**Outcomes:**

Following therapeutic interventions, the patient demonstrated marked improvement in hyponatremia and rhabdomyolysis, culminating in discharge on the twelfth hospital day.

**Lessons:**

Physicians should maintain a high index of suspicion for excessive water intake in patients with urolithiasis who exhibit anxiety, fear, or a history of psychiatric disorders because such behavior can lead to life-threatening electrolyte imbalances and associated complications. Such behaviors may be mitigated or prevented by appropriate medical advice and psychological interventions.

## 1. Introduction

Most cases of primary polydipsia are psychogenic in nature, with few cases of non-psychogenic primary polydipsia reported in the literature.^[1]^ Primary polydipsia commonly occurs in patients with neurodevelopmental disorders, such as autism and intellectual disabilities, as well as chronic psychotic disorders, such as schizophrenia, schizoaffective disorder, bipolar disorder, and psychotic depression.^[2]^ Individuals with nonpsychotic Axis I psychiatric disorders may also experience primary polydipsia, which is often termed compulsive water drinking or psychogenic polydipsia.^[3]^ Additionally, some patients develop primary polydipsia due to excessive concern for dehydration or the erroneous belief that excessive water intake promotes health, even in the absence of a diagnosed psychiatric disorder.^[4]^ This report presents a clinical case of a patient with both ureteral stones and mental disorder who developed hyponatremia and rhabdomyolysis as a result of excessive water intake to facilitate stone passage. The patient’s excessive drinking behavior stems from anxiety and fear associated with mental disorders, exacerbated by excessive adherence to medical advice (“increase fluid intake to facilitate stone passage”), involving both psychological and nonpsychological factors.

## 2. Case presentation

### 2.1. Case history and examination

The patient was a 43-year-old male who presented with left-sided renal colic-like pain approximately 1 month previously ultrasound performed 3 days prior revealed left ureteral stones with mild left hydronephrosis. Hospitalization and surgical intervention were recommended; however, the patient declined owing to surgical anxiety. The physician provided reassurance and advised on increased fluid intake to facilitate stone passage. The patient subsequently increased his daily water intake to 4 to 6 L, which led to the development of facial and bilateral lower extremity edema, along with generalized fatigue, ultimately necessitating hospitalization. The patient had a 30-year history of a low mood and insomnia. He was diagnosed with major depressive disorder at the Yunnan Provincial Psychiatric Hospital and treated with quetiapine fumarate, trazodone hydrochloride, and escitalopram oxalate; however, his symptoms demonstrated minimal improvement. Over the past 6 months, the patient exhibited an elevated mood and racing thoughts and sought treatment at the same psychiatric hospital, where he was diagnosed with bipolar disorder. The patient had a history of multiple suicide attempts and self-inflicting wrist lacerations. Over the past year, he had been maintained on a regimen of quetiapine fumarate, trazodone hydrochloride, escitalopram oxalate, fluvoxamine maleate, zaleplon, and alprazolam. Additionally, the patient had chronic hepatitis B and was receiving tenofovir and hepatoprotective therapies. He had undergone a 1-year course of interferon therapy, which had been discontinued 3 months prior. On physical examination, the patient appeared anxious, had a low mood, and spoke minimally but was fully alert and oriented. Physical examination revealed facial and bilateral lower extremity edema with tenderness and percussion pain in the left renal region. No other significant abnormalities were observed and the patient’s vital signs remained stable. Cardiovascular, respiratory, abdominal, and neurological examinations were unremarkable. Table 1 outlines the timeline of this case report.

**Table 1:** Timeline of the case report.

Time point	Clinical features	Investigations	Management
1 mo prior to admission	Left-sided renal colic-like pain	None	None
3 d prior to admission	Worsening of left-sided renal colic-like pain	Ultrasound: Left ureteral stone and left renal hydronephrosis	Emotional support and advised increased fluid intake
Within 3 d prior to admission	Daily fluid intake of approximately 4–6 L, with progressive facial and bilateral lower extremity edema and generalized weakness	None	None
Day of admission	Left-sided renal colic-like pain, facial and bilateral lower extremity edema, generalized weakness	Sodium: 126.7 mmol/L; Potassium: 3.38 mmol/L Creatine Kinase: 1728 U/L; Lactate Dehydrogenase: 424 U/L Serum Creatinine: 139 µmol/L; Urea: 6.73 mmol/L Albumin: 30.4 g/L; AST: 78 U/L Hemoglobin: 92 g/L; RBC: 3.88 × 10¹²/L HBsAg: Positive; HBeAb: Positive; HBcAb: Positive Urine Red Blood Cells (HPF): 9.25/HPF Urine White Blood Cells (HPF): 4.86/HPF Abdominal CT: Left-sided ureteral stone in the bladder wall segment, left renal pelvis and ureteral dilatation Cardiac Ultrasound: Left ventricular diastolic dysfunction, grade I	None
Day 2	Palpitations, shortness of breath, occasional left-sided renal colic-like pain	Sodium: 128.5 mmol/L; Potassium: 3.87 mmol/L Creatine Kinase: 2206 U/L; Lactate Dehydrogenase: 475 U/L Serum Creatinine: 132 µmol/L; Urea: 6.98 mmol/L	Intravenous Furosemide 20 mg bolus
Day 3	Palpitations and shortness of breath improved compared to the previous day	Sodium: 130.2 mmol/L; Potassium: 3.96 mmol/L Creatine Kinase: 2077 U/L; Lactate Dehydrogenase: 436 U/L Serum Creatinine: 129 µmol/L; Urea: 7.89 mmol/L	Furosemide 60 mg via continuous infusion Furosemide 40 mg via continuous infusion
Day 4	Palpitations and shortness of breath significantly improved, edema reduced	Midstream urine culture showed Escherichia coli, sensitive to Cefoperazone	20% Human albumin 10 mg intravenous infusion + 0.9% NaCl 100 mL intravenous infusion Furosemide 20 mg IV bolus Dopamine 20 mg via continuous infusion Furosemide 40 mg via continuous infusion Quetiapine Fumarate 25 mg oral (until discharge) Sertraline 25 mg oral (until discharge)
Day 5	Condition stable	Sodium: 134.2 mmol/L; Potassium: 3.30 mmol/L Creatine Kinase: 1356 U/L; Lactate Dehydrogenase: 420 U/L Serum Creatinine: 129 µmol/L; Urea: 5.19 mmol/L Albumin: 31.9 g/L; AST: 67.1 U/L Hemoglobin: 87 g/L; RBC: 3.65 × 10¹²/L	In addition to Day 4 treatment: Furosemide 40 mg continuous infusion (morning dose) Potassium Chloride sustained-release tablets 1 g/dose, bid, oral (until discharge)
Day 6	Nighttime abdominal pain, edema nearly resolved	Follow-up abdominal CT: Suspected mid-abdominal peritonitis; other findings similar to prior results	In addition to Day 4 treatment: Cefoperazone 1 g IV + 0.9% NaCl 100 mL intravenous infusion Split Furosemide 40 mg continuous infusion into 2 doses of 20 mg each Discontinue Dopamine
Day 7	Condition stable	None	Furosemide 20 mg IV bolus qid Cefoperazone 1 g IV + 0.9% NaCl 100 mL intravenous infusion
Day 8	Condition stable	None	Same as Day 7
Day 9	Condition stable	Sodium: 140.8 mmol/L; Potassium: 3.26 mmol/L Creatine Kinase: 420 U/L; Lactate Dehydrogenase: 328 U/L Serum Creatinine: 128 µmol/L; Urea: 5.40 mmol/L Albumin: 29.5 g/L; AST: 30.3 U/L Hemoglobin: 71 g/L; RBC: 3.03 × 10¹²/L WBC: 2.69 × 10⁹/L; Platelets: 92 × 10⁹/L	Furosemide 20 mg IV bolus qid Cefoperazone 1 g IV + 0.9% NaCl 100 mL intravenous infusion Presurgical preparation
Day 10	Condition stable	None	Ureteroscopic lithotripsy
Day 11	Condition stable	Sodium: 137.9 mmol/L; Potassium: 4.16 mmol/L Creatine Kinase: 227 U/L; Lactate Dehydrogenase: 357 U/L Serum Creatinine: 103 µmol/L; Urea: 6.22 mmol/L Albumin: 30.5 g/L; AST: 27.1 U/L Hemoglobin: 73 g/L; RBC: 3.12 × 10¹²/L; Platelets: 99 × 10⁹/L Abdominal CT: Post-left ureteral stent placement Cranial CT: Mild brain atrophy	Furosemide 20 mg IV bolus tid
Day 12	Condition stable, with no significant discomfort	None	Discharge arranged with relevant medical advice: Fluid intake restriction, 1 L/d

AST = aspartate aminotransferase, CT = computed tomography, HPF = high power field, IV = intravenous, RBC = red blood cell.

### 2.2. Investigation and management

On admission, laboratory tests revealed that the patient had hypotonicity (274.86 mOsm/kg H₂O, calculated using the formula: plasma osmolality = 2 × [sodium (mmol/L) + potassium (mmol/L)] + glucose (mmol/L) + urea (mmol/L)), hyponatremia (126.7 mmol/L), and hypokalemia (3.38 mmol/L). Creatine kinase (CK) was significantly elevated to 1728 U/L, lactate dehydrogenase to 424 U/L, serum creatinine to 139 µmol/L, and aspartate aminotransferase to 78.0 U/L, all of which were above the normal range. The patient’s serum albumin level was 30.4 g/L, hemoglobin level was 92 g/L, and red blood cell count was 3.88 × 10¹²/L, indicating anemia. Tests for hepatitis B surface antigen, core antibody, and e-antibody were positive, indicating chronic hepatitis B infection. Urinalysis showed 9.25/HPF red blood cells and 4.86/HPF white blood cells, suggesting possible urinary tract infection and abdominal computed tomography revealed a left-sided ureterovesical junction stone, along with a left renal pelvis and ureteral hydronephrosis. Echocardiography revealed grade I diastolic dysfunction, indicating cardiac insufficiency. Due to limitations in the hospital’s laboratory capabilities, measurements of urine osmolality, urinary sodium, and urine specific gravity were not obtained at the time of admission. Despite the absence of these key parameters, the diagnosis of dilutional hyponatremia (also referred to as water intoxication or hypervolemic hypotonic hyponatremia) was supported by the patient’s low serum sodium and plasma osmolality, clinical signs of hypervolemia (notably facial and bilateral lower extremity edema), and a clear precipitating factor of excessive water intake to promote stone passage. Furthermore, the concurrent elevations in CK and lactate dehydrogenase levels confirmed the presence of rhabdomyolysis as a complication.

The patient was started on furosemide for diuresis on the second day of hospitalization to alleviate edema and reduce the cardiac load. The dose of furosemide was gradually increased, and it was switched to continuous infusion via a micropump for more precise control. On the fourth day, as edema persisted without significant reduction, it was suspected that the edema might also be related to the liver dysfunction and hypoalbuminemia secondary to chronic hepatitis B. Human albumin was administered to increase colloid oncotic pressure and further reduce edema. To enhance renal perfusion, low-dose dopamine was administered using a micropump. Additionally, given the patient’s psychiatric symptoms, quetiapine fumarate and sertraline hydrochloride were administered for symptomatic treatment. As the treatment progressed, the patient’s palpitations and dyspnea gradually improved, and the edema markedly reduced. Urine output increased from 880 to 2600 mL/d. As the condition improved, human albumin and dopamine were gradually discontinued, while furosemide was continued with a decreasing dose. On the sixth day of hospitalization, urine culture and sensitivity testing confirmed a urinary tract infection, and cefoperazone was initiated to treat the infection. On the 10th day, the patient’s condition had significantly improved: edema had resolved, and there were no notable symptoms. After completing preoperative preparations, the patient successfully underwent ureteroscopic lithotripsy with good postoperative recovery.

### 2.3. Outcome

On the eleventh day of admission, follow-up laboratory tests showed that the levels of sodium (137.9 mmol/L), potassium (4.16 mmol/L), serum creatinine (103 µmol/L), and aspartate aminotransferase (27.1 U/L) had returned to normal, and both CK (227 U/L) and lactate dehydrogenase (357 U/L) had decreased to near-normal levels, indicating clinical improvement. The patient was discharged on the 12th day without any significant discomfort, with instructions to restrict fluid intake to 1 L/d. A follow-up visit was scheduled for 1 month to remove the ureteral stent and further assess the patient’s condition.

## 3. Discussion

The earliest case report in the literature on excessive water intake leading to hyponatremia and rhabdomyolysis was published in 1979. Browne PM first reported a case involving an elderly patient who drank excessive amounts of water to increase urine output because he believed that he had a low urine volume.^[5]^ In 1987, Mor F et al reported the case of a 64-year-old patient with severe depression who developed self-induced water intoxication complicated by rhabdomyolysis.^[6]^ In 2016, Lee LC et al reported a case of a 59-year-old woman who developed symptomatic acute hyponatremia due to water intoxication after following the common advice to increase fluid intake for a urinary tract infection.^[7]^ In 2022, Chiang TH et al reported a case in which a patient developed acute hyponatremia and subsequently experienced a seizure after consuming an excessive amount of water during bowel preparation for a colonoscopy.^[8]^ In 2023, Bafarat AY et al reported a case of a 16-year-old male with delusional disorder who developed hyponatremia and subsequent seizures after consuming up to 15 L of water per day.^[9]^ In these cases, excessive water intake was solely due to either psychological or nonpsychological factor. However, our case report describes a 43-year-old male patient with a mental disorder who developed hyponatremia and rhabdomyolysis after attempting to promote the passage of a ureteral stone by drinking excessive amounts of water. The patient’s excessive drinking behavior was attributed to anxiety and fear associated with mental disorders, compounded by the excessive following of medical advice (“increase fluid intake to facilitate stone passage”), involving both psychological and nonpsychological factors.

Hyponatremia is one of the most common electrolyte disturbances encountered in hospitalized patients, and can lead to severe clinical manifestations. Mild symptoms of hyponatremia include impaired concentration, nausea, and gait disturbances; moderate symptoms may present as delirium, confusion, and headache; and severe cases may involve vomiting, seizures, and coma.^[10]^ The treatment of hyponatremia should be tailored based on the severity of the symptoms. Regardless of the duration of hyponatremia, patients with severe symptoms (such as lethargy, seizures, or cardiopulmonary distress) and those with moderate symptoms (such as vomiting and confusion) who are at a high risk of life-threatening complications require emergency treatment. Current guidelines recommend increasing serum sodium levels by 4 to 6 mEq/L (4–6 mmol/L) within 1 to 2 hours, which can reverse hyponatremic encephalopathy.^[10]^ This may be accomplished with an intravenous bolus (central line or peripheral vein) containing a 100 mL or 150 mL dose of 3% sodium chloride, administered over 10 minutes or 20 minutes, which can be repeated two to 3 times until the desired serum sodium level is achieved. For nonemergency patients, treatment should be further adjusted based on volume status. For hypovolemic hyponatremia: Intravenous infusion of isotonic saline or other crystalloid solutions should be administered. Sodium-rich broth and sodium chloride tablets can be used for oral supplementation. Euvolemic hyponatremia: For hyponatremia caused by the syndrome of inappropriate antidiuretic hormone secretion, treatment measures include fluid restriction, increased solute intake (sodium chloride, protein, urea), and the use of vaptans. In patients with hyponatremia due to glucocorticoid deficiency or severe hypothyroidism, hormone therapy with hydrocortisone or levothyroxine should be administered. For hypervolemic hyponatremia: The treatment should target the underlying disease. In heart failure patients, managing hyponatremia requires fluid restriction and medication adjustments to achieve optimal hemodynamic status. Loop diuretics can enhance fluid and electrolyte excretion, thereby reducing fluid overload; however, excessive use may exacerbate hyponatremia. In patients with hyponatremia and cirrhosis, treatment includes fluid restriction, loop diuretics, potassium supplementation, and albumin administration.^[11]^

In this case, based on the patient’s clinical presentation and laboratory findings, the final diagnosis was hypervolemic hypotonic hyponatremia due to excessive water intake. Due to the absence of severe neurological symptoms, emergency treatment was not required. The treatment strategy primarily involved diuresis with furosemide in combination with fluid restriction to gradually eliminate excess water, reduce cardiac workload, and closely monitor electrolyte changes. During the first 72 hours of hospitalization, serum electrolytes were monitored every 24 hours. Subsequent testing was guided by the patient’s clinical condition and treatment needs. Details of serum sodium measurements are provided in Table 1. Based on these results, the dosage of furosemide and fluid restriction measures were appropriately adjusted. At no point did the serum sodium correction rate exceed 4 mmol/L within any 24-hour period, which remained well below the safety threshold of 8 mEq/L/d (8 mmol/L/d) recommended by current clinical guidelines.^[11]^ This approach helped to avoid overly rapid correction of hyponatremia. Too rapid a correction of hyponatremia could cause a rapid shift in plasma osmolality and lead to central or extra pontine myelinolysis.^[12]^ Additionally, psychological interventions were provided in collaboration with psychiatry to alleviate the patient’s anxiety and fear. Albumin was supplemented to correct the hypoalbuminemia caused by chronic hepatitis B, and antibiotics were administered to control urinary tract infection. After adequate correction of hyponatremia and significant clinical improvement, the patient underwent ureteroscopic lithotripsy to relieve the ureteral obstruction caused by the stone, thereby improving renal function and reducing the risk of recurrent excessive water intake triggered by anxiety and fear.

Rhabdomyolysis refers to a clinical syndrome caused by various factors leading to muscle cell necrosis, compromise of cell membrane integrity, and release of intracellular components (such as myoglobin, enzymes, and electrolytes) into the bloodstream, resulting in elevated serum muscle enzymes, electrolyte imbalances, and acute kidney injury.^[13]^ There are numerous causes of rhabdomyolysis, with common causes including trauma, intense muscle activity, infections, drugs/toxins, and electrolyte imbalances (hyponatremia, hypokalemia, hypocalcemia, hypophosphatemia, hypernatremia), as well as some genetic disorders.^[14]^ The mechanism by which hyponatremia and hypokalemia induce rhabdomyolysis remains unclear, but a common pathological mechanism is increased intracellular calcium (Ca^2+^) influx.^[15]^ The clinical manifestations of rhabdomyolysis range from asymptomatic to life-threatening acute kidney injury.^[14]^ The classic triad of symptoms includes muscle pain, fatigue, and dark-colored urine; however, this triad is observed in fewer than 10% of patients.^[16,17]^ Clinically, a CK level greater than 1000 U/L or more than 5 times the upper limit of normal is commonly used as the gold standard for diagnosing rhabdomyolysis.^[18]^ In this case, the patient’s laboratory findings confirmed a diagnosis of rhabdomyolysis. The patient did not present with severe clinical symptoms, which may be attributed to the patient’s early medical consultation and the physician’s timely diagnosis and intervention based on prior clinical experience. This early intervention prevented further muscle cell necrosis and CK elevation. Management of rhabdomyolysis should include treatment of the cause and prevention of its complications such as acute kidney injury. Early and aggressive fluid resuscitation is essential for the prevention of acute kidney injury. However, in this case, due to the presence of water intoxication and hyponatremia, the intensity of fluid resuscitation was relatively low. Instead, diuresis with furosemide was used to maintain adequate urine output and promote the excretion of myoglobin and other nephrotoxic substances, thereby improving renal function.

## 4. Conclusion

In summary, the uniqueness of this case lies in the fact that the patient’s excessive water intake was influenced by both psychological and nonpsychological factors: anxiety and fear associated with mental disorders, as well as excessive adherence to medical advice recommending increased fluid intake to promote stone passage. A limitation of this report is that, due to constraints in hospital laboratory capabilities, urine osmolality and urinary electrolyte levels were not measured. Consequently, there is insufficient laboratory evidence to definitively attribute hyponatremia to excessive water intake. Furthermore, the precise mechanisms by which hyponatremia and hypokalemia induce rhabdomyolysis remain unclear and warrant further investigation. In addition, for patients presenting with both hyponatremia and urinary tract obstruction due to urolithiasis, timely surgical relief of the obstruction may contribute to improved renal function and enhanced urine output, which in turn can facilitate the correction of hyponatremia. However, safe performance of such procedures requires prior management of hyponatremia-related symptoms and complications. The optimal sequence and timing of these interventions should be further explored in future clinical practice.

This case underscores the need for individualized fluid management strategies in patients with urolithiasis who exhibit anxiety, fear, or have a history of psychiatric illness. Physicians should refrain from providing nonspecific recommendations such as simply advising patients to “increase fluid intake” without further clarification. Instead, personalized guidance should include specific recommendations regarding the volume and type of fluid, in order to avoid misinterpretation – such as the belief that more water always leads to better stone clearance or overall health benefits, or misconstruing “fluid” to include sugary drinks, caffeinated beverages, or alcohol, which could disrupt treatment or lead to other metabolic complications. To improve patient compliance and safety, such advice should be provided both verbally and in written form. Furthermore, family members should be actively involved in the patient’s care by monitoring drinking behavior and intake volume. Regular follow-up is essential, with treatment adjustments based on serum sodium levels, body weight, and other clinical indicators. Patients should also be instructed to seek medical attention promptly if symptoms such as impaired concentration, nausea, vomiting, gait disturbances, or headache develop. Furthermore, in patients with hyponatremia or hypokalemia, physicians should be vigilant for rhabdomyolysis as a potential complication because of the risk of causing acute kidney injury and other severe consequences. Serum CK levels should be promptly assessed in patients presenting with hyponatremia or hypokalemia to guide clinical management and prevent serious outcomes.

## Acknowledgments

We are grateful to the medical personnel who were caring for the patient.

## Author contributions

**Conceptualization:** Jian Ji.

**Data curation:** Xiaowen Gao, Lingmin Lei.

**Investigation:** Xiaowen Gao, Yafei Wang, Tiancan Yang.

**Supervision:** Jian Ji, Lvyang Chen, Yuanquan Zhu.

**Writing – original draft:** Xiaowen Gao.

**Writing – review & editing:** Xiaowen Gao, Jian Ji, Yafei Wang, Tiancan Yang, Lingmin Lei, Lvyang Chen, Yuanquan Zhu.
